# Hospitalization and Morbidity Rates After Pediatric Traumatic Brain Injury: A Nation-Wide Population-Based Analysis

**DOI:** 10.3389/fped.2021.747743

**Published:** 2021-09-30

**Authors:** Nora Bruns, Pietro Trocchi, Ursula Felderhoff-Müser, Christian Dohna-Schwake, Andreas Stang

**Affiliations:** ^1^Department of Pediatrics I, Pediatric Intensive Care Medicine, University Hospital Essen, University of Duisburg-Essen, Essen, Germany; ^2^Center for Translational and Behavioural Neurosciences (TNBS), University Hospital Essen, University of Duisburg-Essen, Essen, Germany; ^3^Institute for Medical Informatics, Biometry, and Epidemiology, University Hospital Essen, University of Duisburg-Essen, Essen, Germany; ^4^Department of Epidemiology, School of Public Health, Boston University, Boston, MA, United States

**Keywords:** pediatric traumatic brain injury, epidemiology, incidence rates, mortality, morbidity, hospitalization

## Abstract

**Background:** Even though traumatic brain injury (TBI) is a major cause of morbidity and mortality in children around the globe, population-based and nation-wide data to assess the burden of TBI is scarce.

**Methods:**Based on diagnosis related groups from nation-wide hospital data, we extracted data on all TBI-related hospitalizations in children <18 years in Germany between 2014 and 2018. We calculated crude, age-specific and standardized incidence rates for hospitalizations, imaging, intracranial injury, neurosurgery, and mortality.

**Results:**Out of 10.2 million hospitalizations, we identified 458,844 cases with TBI as primary or secondary diagnosis, resulting in a crude incidence rate of 687/100,000 child years (CY). Age-specific rates of computed tomography were below 30/100,000 CY until the age of 10 years and increased to 162/100,000 CY until 17 years of age. Intracranial injury was diagnosed in 2.7%, neurosurgery was performed in 0.7% of patients, and 0.7% were mechanically ventilated. Mortality was 0.67/100,000 CY (0.1%).

**Conclusions:**Despite substantial hospitalization rates for pediatric TBI in Germany, the rates of imaging, the need for mechanical ventilation, neurosurgery and mortality were overall very low. Reasons for hospitalization and measures to reduce unnecessary admissions warrant further investigation.

## Introduction

Traumatic brain injury frequently occurs in children and represents a relevant cause of pediatric morbidity and mortality worldwide (1, 2). Typically, a bimodal age distribution is reported with infants and adolescents as the most affected groups and male predominance across all ages (1). Incidence rates vary considerably between studies and countries, especially with respect to hospitalization and imaging practices (1). For example, in the United States of America (US), annual incidence rates of TBI have been estimated to be 799/100,000 children (3). Crude annual US hospitalization rates declined from 119/100,000 children below 18 years to ~73/100,000 between the 1990s and 2005 (4).

In European countries, annual standardized hospitalization rates after TBI vary between 81 and 643/100,000 persons (children and adults). With 584/100,000, Germany has the second highest annual hospitalization rate in Europe but with a rather low mortality of 8.3/100,000 (2). For children, population-based data on the hospitalization rates for TBI, TBI-related morbidities, and mortality are scarce. The Collaborative Pediatric TBI Working Group recently identified epidemiologic data to assess the burden of pediatric TBI as one of the key clinical research needs in this field (5).

The aim of this study was to calculate nation-wide population-based rates of pediatric hospitalizations for TBI, imaging, treatment, and mortality in Germany. We analyzed a comprehensive data set provided by the Federal Bureau of Statistics that contains information on all hospitalizations of patients <18 years in German hospitals between 2014 and 2018.

## Methods

Since 2004, imbursement for German hospitals is based on diagnosis related groups (DRG). By law (§21 KHEntgG), German hospitals must transmit data on all hospitalizations to the Hospital Remuneration System (InEK). After plausibility control, data are anonymized and forwarded to the Federal Bureau of Statistics. As the submission of hospitalization data is mandatory for reimbursement, hospitals have a strong incentive to supply a comprehensive data set.

For this study we analyzed 10,244,649 hospitalizations of patients <18 years between 2014 and 2018 from all hospitals in Germany. Information on the structure of the DRG data set is supplied by the Federal Bureau of Statistics and has been described in detail elsewhere (6–8). More information is available on https://www.forschungsdatenzentrum.de/en/health/drg.

### Data Extraction and Calculation of Variables

We extracted cases with primary or secondary diagnosis of traumatic brain injury (TBI) according to the International Classification of Diseases, 10th Edition, German Modification (ICD-10-GM) (ICD-Code: S06). To assess the clinical course, we extracted information on secondary diagnoses and procedures based on ICD codes and codes for surgeries and procedures (Operations- und Prozedurenschlüssel, OPS) (Supplementary Table 1). New variables calculated based on ICD and OPS codes were ventilation, resuscitation, imaging, intracranial injury, combination of neurosurgery, complications (Supplementary Table 1).

### Population Data and Standardization

We extracted data of the end-of-year populations for each 1-year age group until age group 17.0–17.9 years between 2013 and 2018 from the homepage of the Federal Bureau of Statistics (https://www.destatis.de/DE/Themen/Gesellschaft-Umwelt/Bevoelkerung/Bevoelkerungsstand/_inhalt.html) and calculated mid-year populations for 2014 to 2018. WHO World Standard Population 2000–2025 (9), European Standard Population 2011–2020 (10), and the U.S. Standard Population 2000 (11) were used for direct age standardization.

### Primary and Secondary End Points

The unit of analysis was hospital admission for/with TBI. We calculated crude and age-specific incidence rates and standardized incidence rates for TBI-associated hospitalizations, in-hospital mortality, diagnostic imaging (in the emergency department and during the hospital stay), intracranial injuries, neurosurgery, visceral surgery, and complications in patients with TBI.

### Missing Data

There were no missing data on age and primary diagnoses. Missing data on secondary diagnoses and procedures were impossible to detect as we could not distinguish whether the diagnosis was not present or whether the diagnosis was just not coded. We assumed that all diagnoses and procedures that are well-reimbursed were comprehensively coded and focussed on these codes for data extraction.

### Software

All calculations were carried out using SAS release 9.4 and SAS Enterprise Guide 7.1 (SAS Institute, Cary, North Carolina, USA).

## Results

Out of 10,244,649 million hospitalizations <18 years of age, we identified 458,844 cases with TBI as primary or secondary diagnosis. TBI was the primary diagnosis in 418,603 cases (91.2%, crude incidence rate 687/100,000 child years) (Table 1). Male patients accounted for 254,032 cases (55.4%) and predominated at all ages except at 14 and 15 years (Supplementary Table 2). The median age was 6 years (10th and 90th percentiles: 0 and 16 years) and the median duration of hospital stay was 2 days (10th and 90th percentiles: 1 and 2 days). Three thousand one hundred eighty-four cases (0.7%, 5/100,000 child years) received mechanical ventilation with a median duration of 22 h (10th and 90th percentiles: 2 and 352 h).

**Table 1:** Clinical information.

	**Overall [*n* (%)]**
Hospitalized with diagnosis of TBI	458,844 (100)
TBI main diagnosis	418,603 (91.2)
Male	254,032 (55.4)
Female	204,812 (44.6)
Age [mean ± SDmedian (10th and 90th percentiles)]	7.1 ± 5.7 6 (0-16)
Length of stay [mean ± SDmedian (10th and 90th percentiles)]	2 ± 3.9 2 (1–2)
Mechanical ventilation	3,184 (0.7)
Mechanical ventilation (hours)[mean ± SDmedian (10th and 90th percentiles)]	120.4 ± 247.2 22 (2–352)
Died	447 (0.1)
Resuscitation	266 (0.1)
Loss of consciousness	64,692 (14.1)
Imaging (CT or MRI)	47,731 (10.4)
CT only	28,361 (6.2)
MRI only	14,864 (3.2)
CT and MRI	4,506 (1.0)
Intracranial injury	12,289 (2.7)
Subdural hemorrhage	4,314 (0.9)
Epidural hemorrhage	2,436 (0.5)
Subarachnoidal hemorrhage	2,154 (0.5)
Brain edema	1,250 (0.3)
Other intracranial injury	6,533 (1.4)
Neurosurgery	3,047 (0.7)
EVD	1,060 (0.2)
Evacuation of hematoma	1,312 (0.3)
DC	627 (0.1)
**Combinations of neurosurgery**	
EVD + evacuation of hematoma	139 (0.0)
EVD + DC	362 (0.1)
DC + evacuation of hematoma	146 (0.0)
Visceral surgery	1,062 (0.2)
Seizures^*^	3,563 (0.8)
Epileptic state	223 (0.0)
Complication(s)	1,468 (0.5)
Early transfer to other hospital	2,408 (0.3)

*TBI, traumatic brain injury; SD, standard deviation; CT, computed tomography; MRI, magnetic resonance imaging; EVD, external cerebrospinal fluid drainage and/or invasive ICP monitoring; DC, decompressive craniectomy*;

* *including any epilepsia*.

**Figure 1:**
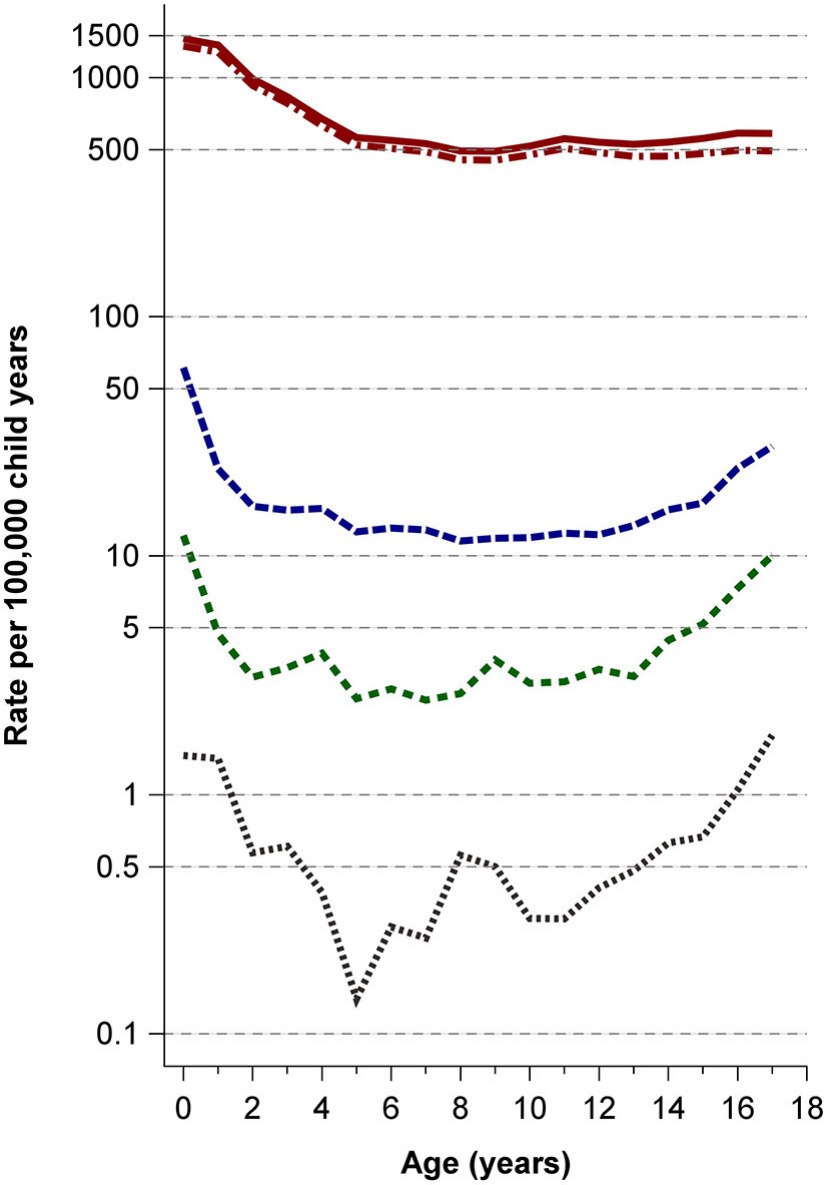
Rates of hospitalizations, TBI main diagnosis, intracranial injury, neurosurgery, and mortality. *Solid red line*: hospitalizations with TBI; *dash-dotted red line*: TBI main diagnosis; *medium-dashed blue line*: intracranial injury; *short-dashed green line*: neurosurgery; *dotted black line*: mortality.

**Figure 2:**
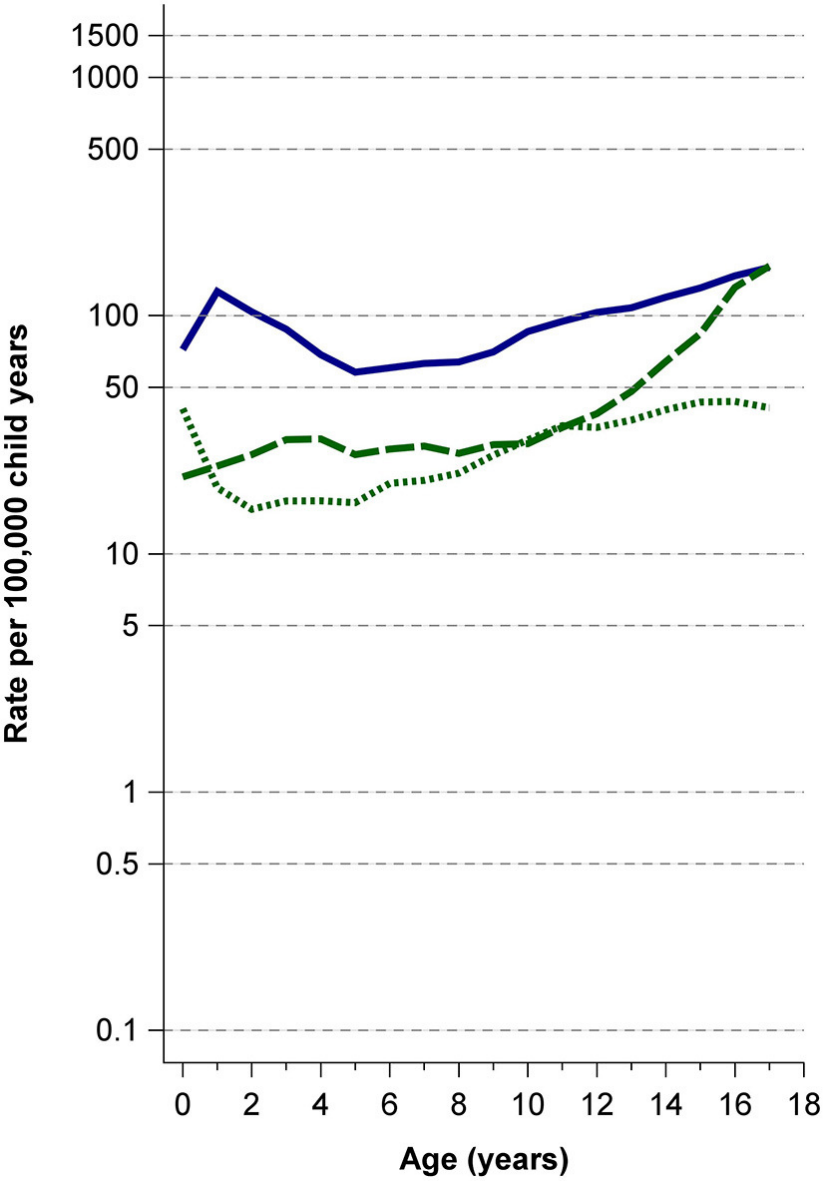
Rates of loss of consciousness and imaging. *Solid blue line*: loss of consciousness; *long-dashed green line*: cranial computed tomography; *short-dashed green line*: cranial magnetic resonance imaging.

Case numbers and incidence rates of all variables varied substantially across ages [Supplementary Table 2 and raw data file at Mendeley Data (12)]. In general, case numbers and age-specific rates were highest among infants and adolescents for hospitalization due to TBI, loss of consciousness, intracranial injury, neurosurgery, and mortality (Figures 1, 2). Hospitalization rates were highest for infants during the first year of life with 1,457/100,000 child years. Imaging by computed tomography was rarely performed up to the age of 10 years and showed a marked increase starting at age 11 (Figure 2; Supplementary Table 2). Imaging (CT/MRI) was performed in 10.4%, intracranial injury was diagnosed in 2.7%, and neurosurgery performed in 0.7% (Table 1). The age-specific incidence rates of visceral surgery increased with age but were generally very low (*n* = 1,062, overall rate 0.2%) (Table 1; Supplementary Table 2). Age-standardized incidence rates for all variables are presented in Table 2.

**Table 2:** Crude and standardized incidence rates.

	**Incidence rates (/100,000 person years) and 95% confidence intervals**			
	**Crude**	**Standardized**		
		**WHO 2020 standard population**	**European standard population**	**U.S. standard population**
TBI hospitalizations	687.1	689.2 (687.2–691.2)	688.0 (686.0–690.0)	681.7 (679.8–683.7)
TBI main diagnosis	626.8	629.7 (627.8–631.6)	628.4 (626.5–630.3)	622.5 (620.6–624.4)
Male	739.4			
Female	631.7			
Mechanical ventilation	4.8	4.7 (4.5–4.8)	4.7 (4.5–4.8)	4.6 (4.5–4.8)
Mortality	0.67	0.65 (0.59–0.71)	0.66 (0.60–0.72)	0.65 (0.59–0.71)
Resuscitation	0.39	0.39 (0.35–0.44)	0.39 (0.35–0.44)	0.39 (0.34–0.43)
Loss of consciousness	96.9	95.5 (94.7–96.2)	95.7 (94.9–96.4)	95.4 (94.6 – 96.1)
Intracranial injury	18.4	18.2 (17.9–18.6)	18.2 (17.9–18.6)	18.0 (17.7–18.4)
Subdural hemorrhage	6.5	6.4 (6.2–6.6)	6.4 (6.2–6.6)	6.3 (6.1–6.5)
Epidural hemorrhage	3.6	3.6 (3.5–3.8)	3.6 (3.5–3.8)	3.6 (3.5–3.7)
Subarachnoidal hemorrhage	3.2	3.1 (3.0–3.3)	3.2 (3.0–3.3)	3.1 (3.0–3.3)
Brain edema	1.9	1.8 (1.7–1.9)	1.8 (1.7–1.9)	1.8 (1.7–1.9)
Other intracranial injury	9.8	9.7 (9.4–9.9)	9.7 (9.4–9.9)	9.6 (9.4–9.8)
Neurosurgery	4.6	4.5 (4.3–4.6)	4.5 (4.3–4.6)	4.4 (4.3–4.6)
EVD	1.6	1.6 (1.5–1.6)	1.6 (1.5–1.7)	1.5 (1.5–1.6)
Evacuation of hematoma	2.0	1.9 (1.8–2.1)	1.9 (1.8–2.1)	1.9 (1.8–2.0)
DC	0.94	0.92 (0.85–0.99)	0.92 (0.85–0.99)	0.91 (0.84–0.99)
Visceral surgery	1.6	1.5 (1.4–1.6)	1.5 (1.4–1.6)	1.5 (1.4–1.6)
Imaging	71.5	69.2 (68.6–69.9)	69.7 (69.1–70.3)	69.5 (68.8–70.1)
CT only	42.5	40.7 (40.2–41.2)	41.1 (40.6–41.6)	40.8 (40.3–41.3)
MRI only	22.3	21.9 (21.6–22.3)	22.0 (21.6- 22.3)	22.1 (21.7–22.4)
CT and MRI	6.7	6.6 (6.4–6.8)	6.6 (6.4–6.8)	6.6 (6.4–6.8)
Seizures^*^	5.3	5.3 (5.1–5.4)	5.3 (5.1–5.4)	5.3 (5.1–5.4)
Epileptic state	0.33	0.33 (0.29–0.38)	0.33 (0.29–0.38)	0.32 (0.28–0.37)

*TBI, traumatic brain injury; SD, standard deviation; CT, computed tomography; MRI, magnetic resonance imaging; EVD, external cerebrospinal fluid drainage and/or invasive ICP monitoring; DC, decompressive craniectomy*;

* *including any epilepsy*.

## Discussion

In this population-based nation-wide study on pediatric TBI, Germany has high hospitalization rates compared to international data (1, 4). Our findings with respect to age and sex distribution concur with published data: Infants had the highest incidence rates of hospitalization for TBI, and males clearly predominated at almost all ages. The rates of imaging, the need for mechanical ventilation, neurosurgery and mortality were overall very low, with highest rates in infants and adolescents. Rates of computed tomography were very low, continuously rising during adolescence.

Mortality, intracranial injuries, neurosurgery, and complications occurred in only a small fraction of patients. While the incidence rates of hospitalizations for TBI in adolescents align with the rates reported for the entire German population, mortality in the pediatric subset was very low compared to the previously published population-wide mortality (2). However, our finding is limited by the fact that it does not include pre-hospital deaths, which account for up to 88% of TBI-related deaths in children (13). The high hospitalization rates and low rates of TBI-related morbidity in our study suggest that not all admitted patients may have required a hospital stay. We assume that computed tomography was avoided at the cost of hospitalizations and patient observation to clinically rule out serious head injury. This inevitably leads to the question of how to identify patients at need for monitoring without hospitalizing healthy children or exposing them to unwarranted radiation.

Several studies tried to account for this dilemma by establishing clinical rules to guide imaging practice. Among three of the most relevant rules (CHALICE, CATCH, and PECARN), PECARN performed best at ruling out clinically important TBI (14–18). In a prospective multicentre validation study, 5.1% of patients attending the emergency department after head trauma had imaging performed and 0.6% of all patients were diagnosed with clinically important TBI (19). The sensitivity for the detection of clinically important TBI was 100% with a specifity of 70%. Even more importantly, the negative predictive value was 100% (19). By ruling out clinically important TBI, the PECARN rule can help to reduce imaging rates. We assume that applying this rule could reassure physicians and parents of children classified into the very low risk group, thereby also lowering hospitalization rates and reducing the social and economic burdens of unwarranted hospitalizations.

However, an approach like the PECARN rule will be difficult to implement in Germany, because the current German DRG system drives hospitals to admit their patients for at least one or two nights to avoid cuts in reimbursement. An additional source leading to hospitalizations may be the lack of a valid German guideline on pediatric TBI and vague criteria for hospitalization after head injury in the expired guideline (20). In the future, clearly phrased criteria for hospitalization after head injury and advances in neuroimaging, like e.g., rapid sequence MRI, could help to identify patients with no need for a hospital stay.

Our study has several limitations owed to the lack of clinical information provided in the DRG data set. Due to the lack of exact information on the causes for admission and death, we likely overestimated these rates. The duration of hospital stay due to TBI may have been shorter than estimated in this study. However, the strength of this study is its comprehensiveness. We analyzed population-based data on TBI-related hospitalizations of children during the 5-year-study period, thereby completely ruling out selection bias and providing nation-wide representative data for Germany.

This study addresses one of the key clinical research needs in the field of pediatric TBI by providing nation-wide comprehensive data on hospitalizations and TBI-related morbidity in Germany. It further provides evidence that hospitalization rates for TBI in Germany were higher in children compared to other countries inside and outside of Europe, with very low rates of mechanical ventilation, intracranial injury, neurosurgery, and mortality. Our data highlight the need to clarify whether TBI-related hospitalizations are determined by actual need for treatment or by reimbursement-related reasons. It should be further explored if there is the potential for quality improvement in the German health care system to reduce a higher than needed hospitalization rate.

## Data Availability Statement

The data analyzed in this study is subject to the following licenses/restrictions: the original dataset can be accessed after inquiry to the Federal Bureau of Statistics of Germany. Requests to access these datasets should be directed to https://www.forschungsdatenzentrum.de/de.

## Ethics Statement

Ethical review and approval was not required for the study on human participants in accordance with the local legislation and institutional requirements. Written informed consent from the participants' legal guardian/next of kin was not required to participate in this study in accordance with the national legislation and the institutional requirements.

## Author Contributions

AS, CD-S, and NB: study design. AS, CD-S, NB, and PT: statistical calculations and verification. NB: drafting of the manuscript. AS, CD-S, NB, PT, and UF-M: critical review and editing of the manuscript. All authors contributed to the article and approved the submitted version.

## Funding

NB received an internal research grant from the Medical Faculty of the University of Duisburg-Essen (IFORES) and a grant from the Stiftung Universitätsmedizin Essen that enabled the conduction of this work.

## Conflict of Interest

The authors declare that the research was conducted in the absence of any commercial or financial relationships that could be construed as a potential conflict of interest.

## Publisher's Note

All claims expressed in this article are solely those of the authors and do not necessarily represent those of their affiliated organizations, or those of the publisher, the editors and the reviewers. Any product that may be evaluated in this article, or claim that may be made by its manufacturer, is not guaranteed or endorsed by the publisher.
