# Supplementary material for: Hospitalization and Morbidity Rates After Pediatric Traumatic Brain Injury: A Nation-Wide Population-Based Analysis
Source: Front Pediatr. 2021 Sep 30;9:747743. doi: 10.3389/fped.2021.747743 (PMC8515415; doi:10.3389/fped.2021.747743)
Supplement: Supplementary file 1 [file Table_1.docx]

Supplementary Table 1: ICD- and OPS-Codes

| **Diagnoses** | **ICD-10-Code(s)** |
| --- | --- |
| Traumatic brain injury | S06 (primary or secondary diagnosis) |
| TBI main diagnosis | S06 (primary diagnosis) |
| Brain edema | S06.1 |
| Epidural hemorrhage | S06.4 |
| Subdural hemorrhage | S06.5 |
| Subarachnoidal hemorrhage | S06.6 |
| Other brain injury (diffuse injury, circumscribed injury, otherwise not specified injury) | S06.2, S06.3, S06.8, S06.9 |
| Loss of consciousness | S06.7 |
| Cardiac arrest | I46, U69.13 |
| Early complication of trauma | T.79 |
| Shock (cardiogenic, hypovolemic, septic) | R57.0, R57.1, R57.2 |
| Sepsis | A40, A41 |
| Acute kidney failure | N17 |
| Pneumonia | J13, J14, J15, J16, J18, J80 |
| Disseminated intravasal coagulation | D65 |
| Seizure (including epilepsy) | G40 |
| Epileptic State | G41 |
|  |  |
| **Procedures** | **OPS-Codes** |
| Cranial computed tomography (CT) | 3-200, 3-220 |
| Cranial magnetic resonance imaging (MRI) | 3-800, 3-820 |
| Neurosurgery | 5-01, 5-02, 5-03, 5-04, 5-05 |
| external cerebrospinal fluid drainage and/or invasive ICP monitoring (EVD) | 5-022.0, 5-029.1 |
| Evacuation of hematoma | 5-013.1, 5-013.4, 5-012.2 |
| Decompressive craniectomy (DC) | 5-021.0, 5-010.1, 5-010.4 |
| Visceral surgery (surgery to the pharynx, larynx, intestinal tract, abdominal organs, kidneys, and urinary tract; surgery due to multiple injury or polytrauma) | 5-29, 5-30, 5-31, 5-42, 5-43, 5-44, 5-45, 5-46, 5-47, 5-48, 5-49, 5-50, 5-51, 5-52, 5-53, 5-54, 5-55, 5-56, 5-57, 5-58, 5-59, 5-981, 5-982 |
| Cardiac or cardiopulmonary resuscitation | 8-771 |
|  |  |
| **Other variables extracted from the basic data set** |  |
| Sex | Included in the DRG data set |
| Length of stay (days) | Included in the DRG data set |
| Ventilation (hours) | Included in the DRG data set |
| Death | Reason for discharge = 7 |
| Early transfer | Reason for discharge = 6 or 8 and *days (“tage”) < 2* |
|  |  |
| **Newly calculated variables** | **Variables/Procedures included**  **[categories]** |
| Ventilation (dichotomous) | Ventilation (hours) not missing  [yes, no] |
| Resuscitation | Cardiac arrest, cardiac or cardiopulmonary resuscitation  [yes, no] |
| Imaging | CT, MRI  [CT only, MRI only, CT + MRI, CT or MRI] |
| Intracranial injury | subdural hemorrhage, epidural hemorrhage, subarachnoidal hemorrhage, brain edema, other brain injury  [yes, no] |
| Combined neurosurgery (dichotomous) | external CSF drainage and ICP monitoring, evacuation of hematoma, decompressive craniotomy  [yes, no] |
| Combined neurosurgery (nominal) | EVD, evacuation of hematoma, DC  [EVD + DC, EVD + evacuation, DC + evacuation] |
| Complication | early complication of trauma, shock,  sepsis, acute kidney failure, pneumonia, disseminated intravasal coagulation  [yes, no] |
