# Supplementary material for: Hospitalization and Morbidity Rates After Pediatric Traumatic Brain Injury: A Nation-Wide Population-Based Analysis
Source: Front Pediatr. 2021 Sep 30;9:747743. doi: 10.3389/fped.2021.747743 (PMC8515415; doi:10.3389/fped.2021.747743)
Supplement: Supplementary file 2 [file Table_2.docx]

**Supplementary table 2:** Age-specific rates

|  | **Age (years)** | | | | | | | | | | | | | | | | | |
| --- | --- | --- | --- | --- | --- | --- | --- | --- | --- | --- | --- | --- | --- | --- | --- | --- | --- | --- |
|  | **0** | **1** | **2** | **3** | **4** | **5** | **6** | **7** | **8** | **9** | **10** | **11** | **12** | **13** | **14** | **15** | **16** | **17** |
| Hospitalizations | 1457.3 | 1370.4 | 988.6 | 832.3 | 674.1 | 561.9 | 547.0 | 530.1 | 493.8 | 492.3 | 517.6 | 556.2 | 537.2 | 526.7 | 537.8 | 557.4 | 586.6 | 584.8 |
| TBI main diagnosis | 1355.9 | 1279.5 | 928.1 | 782.0 | 630.4 | 523.2 | 506.3 | 490.1 | 453.6 | 451.8 | 475.9 | 506.4 | 486.2 | 468.8 | 469.6 | 481.7 | 496.9 | 494.1 |
| Male | 1510.5 | 1390.0 | 1018.8 | 889.3 | 745.7 | 643.0 | 624.6 | 620.3 | 576.5 | 580.8 | 615.1 | 664.6 | 624.4 | 557.2 | 519.8 | 527.9 | 595.9 | 616.9 |
| Female | 1401.3 | 1349.7 | 956.7 | 772.2 | 598.6 | 476.2 | 465.1 | 434.7 | 406.4 | 398.8 | 414.5 | 441.5 | 445.0 | 494.5 | 557.0 | 588.9 | 576.6 | 550.0 |
| Mechanical ventilation | 9.3 | 5.5 | 4.1 | 3.8 | 3.4 | 2.9 | 2.9 | 3.1 | 2.6 | 3.4 | 2.8 | 3.0 | 2.9 | 3.6 | 4.7 | 5.8 | 8.5 | 11.8 |
| Died | 1.46 | 1.42 | 0.57 | 0.61 | 0.39 | 0.14 | 0.28 | 0.25 | 0.56 | 0.50 | 0.30 | 0.30 | 0.41 | 0.48 | 0.63 | 0.67 | 1.05 | 1.77 |
| Resuscitation | 1.25 | 0.96 | 0.38 | 0.41 | 0.28 | 0.17 | 0.25 | 0.17 | 0.28 | 0.25 | 0.22 | 0.22 | 0.11 | 0.13 | 0.29 | 0.23 | 0.60 | 0.85 |
| Loss of consciousness | 72.1 | 126.4 | 103.8 | 87.7 | 68.6 | 57.9 | 60.4 | 63.0 | 63.9 | 70.4 | 85.8 | 94.7 | 103.2 | 107.9 | 119.6 | 130.8 | 147.0 | 159.5 |
| Intracranial injury | 61.2 | 23.1 | 16.1 | 15.5 | 15.8 | 12.6 | 13.1 | 12.8 | 11.5 | 11.8 | 11.9 | 12.4 | 12.2 | 13.4 | 15.6 | 16.6 | 23.2 | 28.7 |
| Subdural hemorrhage | 33.4 | 8.2 | 5.0 | 5.1 | 4.7 | 4.2 | 3.9 | 4.1 | 3.3 | 4.1 | 3.4 | 3.3 | 4.0 | 3.9 | 4.4 | 4.6 | 6.8 | 8.5 |
| Epidural hemorrhage | 12.4 | 4.6 | 2.9 | 3.8 | 4.2 | 2.7 | 2.9 | 2.4 | 2.5 | 2.5 | 2.6 | 2.1 | 2.9 | 2.6 | 3.1 | 3.4 | 3.2 | 4.4 |
| Subarachnoidal hemorrhage | 7.1 | 2.5 | 1.7 | 2.1 | 1.9 | 2.1 | 1.8 | 2.0 | 2.0 | 2.1 | 2.0 | 2.3 | 2.3 | 2.8 | 3.1 | 3.4 | 6.6 | 8.9 |
| Brain edema | 3.6 | 2.2 | 1.0 | 1.4 | 1.0 | 0.8 | 1.1 | 1.1 | 1.1 | 1.4 | 1.6 | 1.5 | 1.4 | 1.3 | 1.5 | 2.3 | 3.9 | 4.8 |
| Other intracranial injury | 17.9 | 11.5 | 9.1 | 7.7 | 8.5 | 6.6 | 7.6 | 8.0 | 7.1 | 6.7 | 7.8 | 7.8 | 7.1 | 7.9 | 9.3 | 10.5 | 15.0 | 17.7 |
| Neurosurgery | 12.2 | 4.7 | 3.1 | 3.4 | 3.9 | 2.5 | 2.8 | 2.5 | 2.7 | 3.7 | 2.9 | 3.0 | 3.3 | 3.1 | 4.4 | 5.2 | 7.3 | 10.0 |
| Extraventricular drainage | 3.11 | 1.85 | 1.09 | 1.11 | 1.37 | 0.87 | 0.78 | 0.95 | 0.67 | 1.25 | 1.24 | 1.18 | 1.25 | 1.12 | 1.55 | 2.03 | 2.67 | 3.93 |
| Evacuation of hematoma | 7.5 | 2.0 | 1.3 | 1.7 | 2.0 | 1.3 | 1.3 | 1.1 | 1.4 | 1.5 | 1.2 | 1.0 | 1.6 | 1.4 | 1.7 | 1.8 | 2.4 | 2.9 |
| Decompressive craniectomy | 1.67 | 1.20 | 0.68 | 0.58 | 0.78 | 0.39 | 0.42 | 0.39 | 0.84 | 0.78 | 0.61 | 0.66 | 0.79 | 0.96 | 0.73 | 0.95 | 1.84 | 2.28 |
| Visceral surgery | 1.5 | 1.1 | 0.7 | 0.6 | 0.7 | 0.7 | 0.9 | 0.7 | 0.6 | 0.8 | 1.0 | 1.0 | 0.9 | 1.3 | 1.6 | 2.4 | 4.9 | 6.2 |
| Imaging (CT or MRI) | 52.7 | 36.9 | 36.7 | 41.8 | 42.1 | 38.2 | 42.5 | 43.8 | 43.9 | 49.3 | 53.9 | 63.4 | 66.4 | 78.3 | 95.7 | 118.5 | 162.9 | 188.8 |
| CT only | 21.0 | 23.4 | 26.1 | 30.2 | 30.4 | 26.1 | 27.5 | 28.3 | 26.4 | 28.7 | 29.0 | 34.0 | 38.8 | 48.1 | 64.2 | 84.0 | 131.4 | 161.5 |
| MRI only | 40.7 | 19.0 | 15.4 | 16.7 | 16.7 | 16.4 | 19.8 | 20.4 | 21.8 | 26.0 | 30.1 | 34.5 | 34.0 | 36.4 | 40.3 | 43.4 | 43.6 | 41.1 |
| CT and MRI | 9.1 | 5.4 | 4.8 | 5.1 | 5.0 | 4.3 | 4.8 | 4.9 | 4.4 | 5.3 | 5.2 | 5.1 | 6.3 | 6.3 | 8.9 | 8.8 | 12.1 | 13.8 |
| Early transfer | 6.1 | 3.7 | 3.5 | 3.4 | 2.8 | 2.8 | 2.6 | 2.3 | 2.9 | 2.7 | 2.6 | 2.8 | 3.8 | 3.2 | 3.7 | 4.3 | 5.6 | 5.4 |
| Seizures* | 6.7 | 4.0 | 3.9 | 4.9 | 3.8 | 4.2 | 4.5 | 4.3 | 3.9 | 4.2 | 4.2 | 5.0 | 5.3 | 5.7 | 6.0 | 7.7 | 7.7 | 8.8 |
| Epileptic state | 2.23 | 0.40 | 0.46 | 0.30 | 0.25 | 0.22 | 0.17 | 0.08 | 0.17 | 0.14 | 0.08 | 0.08 | 0.11 | 0.11 | 0.16 | 0.31 | 0.35 | 0.32 |

All numbers in the table are rates per 100,000 person years. * including any epilepsia
